# Introducing a psychiatric genetic cohort of schizophrenia patients and controls from Vietnam

**DOI:** 10.1192/j.eurpsy.2021.2122

**Published:** 2021-08-13

**Authors:** A. Braun, T.V. Nguyen, S. Ripke, P.V. Nguyen, J. Kraft, H.T. Nguyen, T.C. Le, G. Panagiotaropoulou, I.M. Hahne, K. Böge, E. Hahn, T.M.T. Ta

**Affiliations:** 1 Department Of Psychiatry, Charité – Universitätsmedizin Berlin, Campus Mitte, Berlin, Germany; 2 Department Of Psychiatry, Hanoi Medical University, Hanoi, VietNam; 3 Stanley Center For Psychiatric Research, Broad Institute of MIT and Harvard, Cambridge, United States of America; 4 Department Of Psychiatry, Charité – Universitätsmedizin Berlin, Campus Benjamin Franklin, Berlin, Germany

**Keywords:** Vietnam, genetics, schizophrénia

## Abstract

**Introduction:**

Genome-wide association studies (GWAS) have successfully revealed genetic risk variants for schizophrenia (SCZ). However, the vast majority of GWAS largely comprise European samples. As a result, the derived polygenic risk scores (PRS) show decreased predictive power when applied to non-European populations.

**Objectives:**

A long-term scientific cooperation between the Charité Universitätsmedizin Berlin and the Hanoi Medical University aims to address this limitation by recruiting a large genetic cohort of comprehensively phenotyped schizophrenia patients and controls in Vietnam.

**Methods:**

A pilot study was conducted at the Department of Psychiatry of the Medical University Hanoi in 2017. Data collection encompassed i) genome-wide SNP genotyping of 200 schizophrenia patients and 200 control subjects ii) structured interviews to assess symptom severity (PANSS), iii) clinical parameters (e.g. duration of illness, medication) and demography.

**Results:**

SCZ-PRS of the pilot sample (N=400) were generated using different training data sets: i) European, ii) East-Asian and iii) mixed GWAS summary statistics from the Psychiatric Genomics Consortium’s latest discovery sample. Most variance explained was observed using a mixed discovery sample (R^2^liability=0.053, p=3.11*10^-8^, Pd <0.5), followed by PRS based on the East-Asian summary statistics (R^2^liability=0.0503, p=6.78*10^-8^, Pd <1) and the European sample (R^2^liability=0.0363, p = 4.26*10^-6^, Pd <0.01).

**Conclusions:**

With this pilot project we established an efficient recruitment, genotyping and data analysis pipeline. Our results corroborate previous findings indicating that transferability of PRS across populations depends on the ancestral composition of the initial discovery dataset. We therefore aim to expand data collection efforts in the future in order to improve risk prediction across diverse populations.

**Disclosure:**

No significant relationships.

